# Promotive factors associated with internalising symptoms amongst college students during the COVID-19 lockdown in Enugu metropolis, Nigeria

**DOI:** 10.4102/sajpsychiatry.v28i0.1672

**Published:** 2022-02-15

**Authors:** Awoere Chinawa, Ann Aronu, Edmund Ossai, Josephat Chinawa

**Affiliations:** 1 Department of Community Medicine, Faculty of Medical Sciences, Enugu State University College of Medicine, Enugu, Nigeria; 2 Department of Paediatrics, Faculty of Medical Sciences, University of Nigeria, Enugu, Nigeria; 3 Department of Community Medicine, Faculty of Health Sciences, Ebonyi State University, Abakiliki, Nigeria

**Keywords:** COVID-19, anxiety symptoms, depressive symptoms, college adolescents, social support, resilience, Enugu metropolis, Nigeria

## Abstract

**Background:**

The outbreak of the coronavirus disease 2019 (COVID-19) pandemic has caused a high burden of psychological distress amongst adolescents.

**Aim:**

This study aimed to evaluate associations of personal strengths including resilience and social support with internalising symptoms amongst college students during the lockdown in the wake of the COVID-19 pandemic.

**Setting:**

The study population included students from senior and junior college classes in public schools in Enugu metropolis, Nigeria.

**Method:**

A school-based cross-sectional study design was employed for the study. A two-stage sampling technique was used to select 496 students (mean age = 16.5, s.d. = 1.9 years; 52.2% female) in six out of 33 public colleges in Enugu metropolis, Nigeria during the lockdown period occasioned by the COVID-19 pandemic. Validated questionnaires assessing anxiety, depression, resilience and social support were used to collect information.

**Results:**

Most of the students reported depressive symptoms, whilst just over a third of the sample reported anxiety or both depressive and anxiety symptoms. Chi-square and logistic regression analyses revealed that being male and reporting higher levels of social support and the ability to bounce back from stress were associated with less anxiety. Being younger and reporting a moderate level of support were associated with more depressive symptoms, whilst the ability to bounce back was associated with fewer depressive symptoms.

**Conclusions:**

Good social support and the ability to bounce back from stress were linked to lower levels of anxiety and depressive symptoms amongst college adolescents during the lockdown in the wake of the COVID-19 pandemic despite high prevalence rates.

## Introduction

Since the inception of the novel COVID-19 pandemic until the time of writing this article, the 7th of November 2020, 49 373 235 cases of COVID-19 have been reported with 1 243 083 deaths.^1^ In Nigeria, 63 731 cases and 1155 deaths were reported.^2^ To control the pandemic, several control measures were applied in Nigeria with restrictions, including traffic restrictions, school closure and quarantine and self-isolation.^3^ The pandemic has also caused separation from loved ones, family schisms, shortage of food supplies and financial distress because of the loss of jobs.^3^ Although these measures curbed the spread of the disease to some extent, however, there exists tremendous psychological distress, especially amongst the adolescents in this vicinity. The negative impact of COVID-19 on adolescents’ mental health triggered by limited social interaction, school closure, reduced number of daily activities and introduction of the new normal phenomenon cannot be overemphasised.^4^

The burden of anxiety and depressive symptoms arising from the COVID-19 pandemic on adolescents is much greater than that observed in adults.^3,4,5^ This could be because of their vulnerability to stress, developmental and hormonal changes that are prevalent in this age. Furthermore, in the recent severe acute respiratory syndrome (SARS) pandemic, psychiatric morbidities were seen to be associated with younger age.^3,4,5^

Careful search in the literature showed no documented studies in this subject amongst adolescents during the COVID-19 pandemic. For instance, Lei et al.^6^ noted the prevalence rate of depressive symptoms during the epidemic in China to be 14.6%, whilst Wang et al.^7^ documented a prevalence rate of 16.5% in respondents with moderate to severe depressive symptoms. In Italy, Rossi et al.^8^ documented the rate of depressive symptoms during the period of the outbreak as 17.3%. Unfortunately, none of these studies were amongst adolescents.

Resilience is the most potent factor that influences the outcome of adolescents with anxiety and/or depressive symptoms in the event of the COVID-19 pandemic.^9^ Although resilience may include survival and adaptation to challenges, it also includes the ability to recover from negative emotional and traumatic experiences. Resilience is an important determinant of quality of life and is an independent predictor of coping with adverse events.^9^

Social support is a very important concept in the management of adolescents with anxiety and depressive symptoms. It involves material aid and involvement of relations and community participation.^10,11^ Social support can help to control anxiety or depressive symptoms in adolescents during a stressor.^12,13^

There is a link between adequate social support and the prevalence of depressive and anxiety symptoms. For instance, low levels of social support are more likely to lead to depressive and anxiety symptoms when an individual is exposed to stress.^14,15^ In the corollary, higher levels of social support could be a measure in addressing depressive and anxiety symptoms amongst adolescents who experienced the outbreak of COVID-19.

Few studies have documented different degrees of resilience and social support in adults with anxiety and depressive symptoms during the COVID-19 pandemic; however, there is a paucity of documented studies amongst adolescents.^6,7,8,9^ In our locale, adolescents have suffered untold psychological trauma, which often goes unnoticed. Thus, this study aimed to investigate levels of anxiety and depressive symptoms amongst college students and to examine associations between social support and the ability to bounce back and these symptoms in the wake of the COVID-19 lockdown.

## Methods

### Study area

Enugu is the administrative capital of Enugu state, which is one of the five states in the southeast geo-political zone of Nigeria. Enugu metropolis is made up of three local government areas, including Enugu North, Enugu South and Enugu East. The inhabitants of the metropolis are mainly of Igbo ethnic nationality with a mixture of other tribes and are predominantly Christians. There are 314 public secondary schools in Enugu state, of which 33 are in Enugu metropolis.

### Study design

This is a school-based cross-sectional study amongst college adolescents who resumed classes in their various schools during the COVID-19 lockdown.

### Sample size determination

The minimum sample size for the study was determined by the formula used for simple proportions.^16^ A total of 493 respondents were included in the study based on a type 1 error (α) of 0.05, a tolerable margin of error of 0.05 and a proportion of 34.1%, representing the proportion of adolescent college students who had anxiety symptom disorders in a previous study in south-east Nigeria.^17^

### Study population

The study population included students from senior and junior college classes in public schools located in Enugu metropolis, Nigeria. These were students who registered for their junior or senior secondary school certificate examinations in the selected schools. Amidst the COVID-19 lockdown in Nigeria, the students were recalled from their homes to enable them to write their examinations.

#### Inclusion criteria

Adolescents who registered for certificate examinations amidst the COVID-19 lockdown and who gave consent to participate in the study were included.

#### Exclusion criteria

Children with known mental health disorders and adolescents who refused to provide consent to participate in the study were excluded.

### Sampling technique

A two-stage sampling technique was used to select the students for inclusion in the study. There are 33 public secondary schools in Enugu metropolis, which included 14 public secondary schools in Enugu South, nine in Enugu North and 10 in Enugu East local government areas. Using a simple random sampling technique of balloting, two schools were selected from the list of public secondary schools in each of the three local government areas. This served as the first stage.

In the second stage, a list of all students in senior secondary class three and junior secondary class three was made (these two classes were the only ones permitted by the Government of Nigeria to return to school since the closure of the schools because of the COVID-19 pandemic. This was to enable the students to prepare and write their various terminal examinations amidst the COVID-19 pandemic). The number of students in the two classes in the six selected schools from school records was 2023. This served as the sampling frame. Sampling interval was obtained by dividing this number by the sample size of 496; hence, a sampling interval of 4 was used. So, every fourth student was recruited for the study based on the sitting arrangement of the students in their various classes on each day of data collection. The index student on each day of data collection was selected using a simple random sampling technique of balloting. The sample used in this study could be considered to be representative of a larger population because of the probability sampling techniques used in the selection of the respondents. The male students were coded 1, whilst female students were coded 2.

### Study instrument

All questionnaires used in the study were validated and had been used previously in Nigeria.^18,19,20^ The 14-item Hospital Anxiety and Depression Scale (HADS)^18^ was used to assess anxiety symptoms and depressive symptoms amongst the students. Response options were on a four-point Likert scale and included 3 (Yes, definitely), 2 (Yes, sometimes), 1 (No, not much) and 0 (Not at all). Items were recoded such that higher values reflected more anxiety and depressive symptoms, with a possible range of 0–21 for each subscale. The Child and Youth Resilience Measure (12 items) was used to assess the level of resilience amongst the students.^19^ The Oslo three-item social support scale^20^ was used to assess social support amongst the respondents. Information on the socio-demographic characteristics of the respondents was also obtained. The questionnaires have been used in previous studies in Nigeria.^18,19,20^

The socio-economic class of the parents of the respondents was determined using social classification.^21^ This was computed by adding the educational attainment of the mother and the occupation of the father of the respondent based on the scoring system of the scale and dividing the sum by 2. After division, a score of 1 was regarded as a high socio-economic class, and scores of 2 and 3 were regarded as middle socio-economic classes. A score of 4 and 5 placed the respondent in a low socio-economic class.

### Ethical considerations

Ethical approval was sought and obtained from the Ethics and Research committee of the University of Nigeria Teaching Hospital Enugu (IRB No. 00002323). Verbal consent was also granted by the adolescents before they were recruited.

#### Data collection method

The validated questionnaires were administered to the students by trained research assistants. This was maintained for all students in the junior secondary class. The research assistants were individuals who have completed secondary school education. They were trained for two days on data collection using the questionnaires and observance of COVID-19 protocols as explained by the various agencies of government.

#### Results

Table 1 shows the socio-demographic characteristics of the respondents. The mean age of the respondents was 16.5 ± 1.9 years, and the majority of the respondents, 78.6% were in the age group 15–19 years. A higher proportion of the respondents, 52.2%, were females. The majority of the respondents, 80.8%, were in senior secondary, third grade.

**TABLE 1:** Socio-demographic characteristics of respondents.

Variable	Frequency (*n* = 496)	*%*
**Age**		
Mean ± (s.d.)	16.5 ± 1.9	-
**Age of respondents in groups (years)**		
< 15	89	17.9
15–19	390	78.6
≥ 20	17	3.4
**Gender**		
Male	237	47.8
Female	259	52.2
**Class of study**		
Senior secondary three	401	80.8
Junior secondary three	95	19.2
**Living together with parents**		
Yes	372	75.0
No	124	25.0
**Educational attainment of father**		
No formal education	16	3.2
Primary education	141	28.4
Secondary education	84	16.9
Tertiary education	255	51.4
**Employment status of mother**		
Unemployed	42	8.5
Self-employed	253	51.0
Salaried employment	201	40.5
**Socio-economic class of parents**		
High socio-economic class	175	35.3
Middle socio-economic class	165	33.3
Low socio-economic class	156	31.5

Less than half of the respondents (192; 38.7%) had anxiety symptoms. Amongst the respondents, 98 (19.8%) had mild anxiety symptoms, 74 (14.9%) moderate and 20 (4.0%) had severe anxiety symptoms. The majority of the respondents, (307; 61.9%) had depressive symptoms; 156 (31.5%) respondents had mild depressive symptoms, 127 (25.6%) had moderate and 24 (4.8%) had severe depressive symptoms. Amongst the respondents, about a quarter of the respondents, (164; 33.1%) were normal, 25 (5.0%) respondents had anxiety symptoms only, 140 (28.2%) had depressive symptoms only, whilst 167 (33.7%) respondents had both anxiety and depressive symptoms.

Table 2 shows the factors associated with anxiety amongst the respondents. The male respondents were about twice less likely to be anxious when compared with female respondents (adjusted odds ratio [AOR] = 0.6, 95% CI: 0.4–0.9). The respondents who had poor social support were 3.2 times more likely to be anxious when compared with those who had strong social support (AOR = 3.2, 95% CI: 1.8–5.9). The respondents who were resilient were twice less likely to be anxious when compared with those who were not resilient (AOR = 0.5, 95% CI: 0.3–0.7).

**TABLE 2:** Factors associated with anxiety amongst the respondents.

Variable	Anxiety (*n* = 496)				*p*-value on bivariate analysis	AOR	95% confidence interval
	Yes		No				
	*n*	%	*n*	%			
**Age of respondents (years)**							
< 15	28	31.5	61	68.5	0.300	NA	-
15–19	157	40.3	233	59.7	-	-	-
≥ 20	7	41.2	10	58.8	-	-	-
**Gender**							
Male	79	33.3	158	66.7	0.019	0.6	0.4–0.9
Female	113	43.6	146	56.4	-	1	-
**Class of study**							
Senior secondary three	164	40.9	237	59.1	0.040	1.5	0.9–2.5
Junior secondary three	28	29.5	67	70.5	-	1	-
**Socio-economic class of parents**							
High socio-economic class	62	35.4	113	64.6	0.478	NA	-
Middle socio-economic class	69	41.8	96	58.2	-	-	-
Low socio-economic class	61	39.1	95	60.9	-	-	-
**Employment status of mother**							
Unemployed	13	31.0	29	69.0	0.553	NA	-
Self-employed	99	39.1	154	60.9	-	-	-
Salaried employment	80	39.8	121	60.2	-	-	-
**Educational attainment of father**							
Tertiary education	95	37.3	160	62.7	0.494	NA	-
Secondary education and less	97	40.2	144	59.8	-	-	-
**Living with both parents together**							
Yes	137	36.8	235	63.2	0.136	0.7	0.5–1.1
No	55	44.4	69	55.6	-	1	-
**OSLO Social Support Scale**							
Poor social support	44	62.0	27	38.0	< 0.001	3.2	1.8–5.9
Moderate social support	85	39.7	129	60.3	-	1.3	0.9–2.1
Strong social support	63	29.9	148	70.1	-	1	-
**Resilience status of respondent**							
Resilient	94	30.8	211	69.2	< 0.001	0.5	0.3–0.7
Not resilient	98	51.3	93	48.7	-	1	-

AOR, adjusted odds ratio; NA, not applicable.

Table 3 shows the factors associated with depression amongst the respondents. The respondents who were less than 15 years were about five times less likely to be depressed when compared with those who were 21 years and above (AOR = 0.2, 95% CI: 0.03–0.9). The respondents who had moderate social support were 1.6 times more likely to be depressed when compared with those who had strong support (AOR = 1.6, 95% CI: 1.1–2.5). The respondents who were resilient were twice less likely to be resilient when compared with those who were not resilient (AOR = 0.5, 95% CI: 0.3–0.7).

**TABLE 3:** Factors associated with depression amongst the respondents.

Variable	Depression (*n* = 496)				*p*-value on bivariate analysis	AOR	95% confidence interval
	Yes		No				
	*n*	%	*n*	%			
**Age of respondents (years)**							
< 15	41	46.1	48	53.9	0.001	0.2	0.03–0.9
15–19	252	64.6	138	35.4	-	0.3	0.1–1.3
≥ 20	14	82.4	3	17.6	-	-	-
**Gender**							
Male	150	63.3	87	36.7	0.540	NA	-
Female	157	60.6	102	39.4	-	1	-
**Class of study**							
Senior secondary three	261	65.1	140	34.9	0.003	0.9	0.3–2.5
Junior secondary three	46	48.4	49	51.6	-	1	-
**Socio-economic class of parents**							
High socio-economic class	101	57.7	74	42.3	0.284	NA	-
Middle socio-economic class	109	66.1	56	33.9	-	-	-
Low socio-economic class	97	62.2	59	37.8	-	-	-
**Employment status of mother**							
Unemployed	25	59.5	17	40.5	0.946	NA	-
Self-employed	157	62.1	96	37.9	-	-	-
Salaried employment	125	62.2	76	37.8	-	-	-
**Educational attainment of father**							
Tertiary education	159	62.4	96	37.6	0.829	NA	-
Secondary education and less	148	61.4	93	38.6	-	-	-
**Parents living together**							
Yes	220	59.1	152	40.9	0.029	0.6	0.4–0.9
No	87	70.2	37	29.8	-	1	-
OSLO Social Support Scale							
Poor social support	51	71.8	20	28.2	0.003	1.8	0.9–3.4
Moderate social support	143	66.8	71	33.2	-	1.6	1.1–2.5
Strong social support	113	53.6	98	46.4	-	1	-
**Resilience status of respondent**							
Resilient	166	54.4	139	45.6	< 0.001	0.5	0.3–0.7
Not resilient	141	73.8	50	26.3	-	1	

AOR, adjusted odds ratio; NA, not applicable.

Table 4 shows the correlation matrix. There is a weak positive correlation between social support and resilience, increases in resilience correlated with increases in social support and this was found to be statistically significant (*n* = 496, *r* = 0.321, *p* < 0.001). There was a very weak negative correlation between resilience and anxiety symptoms, increases in resilience correlated with decreases in anxiety symptoms and this was found to be statistically significant, (*n* = 496, *r* = –0.269, *p* < 0.001). There was a strong positive correlation between anxiety and depressive symptoms, increases in anxiety symptoms correlated with increases in depressive symptoms and this was found to be statistically significant (*n* = 496, *r* = 0.581, *p* < 0.001).

**TABLE 4:** Correlation matrix.

Variable	*n* = 496	R *p*-value	R *p*-value	R *p*-value	R *p*-value	Mean	s.d.
	1	2	3	4	5		
1.Social support	-	0.321 < 0.001	−0.193 < 0.001	−0.195 < 0.001	0.011 0.809	10.9	2.2
2.Reslience	-	-	−0.262 < 0.001	−0.269 < 0.001	−0.036 0.419	51.6	6.7
3.Depressive symptoms	-	-	-	0.581 < 0.001	0.132 0.003	8.7	3.5
4.Anxiety symptoms	-	-	-	-	0.043 0.342	6.4	4.2
5.Age	-	-	-	-	-	16.5	1.9

s.d., standard deviation.

#### Predictors of anxiety symptoms

Table 5 shows the regression model summary. With the adjusted R^2^ of 0.351, the model predicts that 35.1% of variability in anxiety symptoms is explained by age, social support, resilience and depressive symptoms.

**TABLE 5:** Regression model summary.

Model	*R*	*R* ^2^	Adjusted *R* square	Standard error of the estimate	*f*	*p*
1	0.597	0.356	0.351	3.364	67.896	< 0.001

Table 6 shows the predictors of anxiety symptoms. For a unit change in resilience, anxiety symptoms decrease by 0.069 units and this was found to be statistically significant (B = –0.069, 95% CI: –0.117 to –0.021). Also, for a unit change in depressive symptoms, anxiety symptoms increase by 0.659 units, and this was found to be statistically significant (B = 0.659, 95% CI: 0.569–0.750).

**TABLE 6:** Predictors of anxiety symptoms.

Variable	Unstandardised coefficients		*t*	*p*	95% CI for B	
	B	Std. error			Lower	Upper
Constant	6.497	1.920	3.384	0.001	2.725	10.269
Age	−0.072	0.080	−0.901	0.368	−0.228	0.085
Social support	−0.103	0.074	−1.406	0.160	−0.248	0.041
Resilience	−0.069	0.024	−2.806	0.005	−0.117	−0.021
Depressive symptoms	0.659	0.046	14.324	< 0.001	0.569	0.750

#### Predictors of depressive symptoms

Table 7 shows the regression model summary. With the adjusted R-square of 0.359, the model predicts that 35.9% of variability in depressive symptoms is explained by age, social support, resilience and anxiety symptoms.

**TABLE 7:** Regression model summary.

Model	*R*	*R* ^2^	Adjusted *R*^2^	Standard error of the estimate	*f*	*p*
1	0.603	0.364	0.359	2.769	70.193	< 0.001

Table 8 shows the predictors of depressive symptoms. For one unit change in age, depressive symptoms increase by 0.192 units and this was found to be statistically significant (B = 0.192, 95% CI: 0.064–0.320). For a unit change in resilience, depressive symptoms decrease by 0.049 units and this was found to be statistically significant (B = –0.049, 95% CI: –0.089 to –0.009). Similarly, for one unit change in anxiety symptoms, depressive symptoms increase by 0.447 units and this was found to be statistically significant (B = 0.447, 95% CI: 0.386–0.508).

**TABLE 8:** Predictors of depressive symptoms.

Variable	Unstandardised coefficients		*t*	*p*	95% CI for B	
					Lower	Upper
	*B*	Std. error				
Constant	6.212	1.574	3.946	<0.001	3.119	9.304
Age	0.192	0.085	2.956	0.003	0.064	0.320
Social support	−0.093	0.061	−1.531	0.127	−0.212	0.026
Resilience	−0.049	0.020	−2.421	0.016	−0.089	−0.009
Anxiety symptoms	0.447	0.031	14.324	<0.001	0.386	0.508

## Discussion

This study aimed to document levels of anxiety and depressive symptoms amongst college students in Nigeria in the wake of the COVID-19 lockdown and to examine associations between social support and the ability to bounce back and these symptoms.

The lockdown caused lots of psychological distress amongst adolescents. This is because children need to have access to peers to maintain cognitive and social development. A study has shown a rising incidence of sexual, emotional and physical violence against children during the COVID-19 pandemic lockdown.^22^ Besides, the worsening emotional and health conditions of other family members could affect their adolescent children.

This study has shown associations of resilience and social support in adolescents with anxiety and depressive symptoms. The prevalence rates of anxiety and depressive symptoms found amongst adolescents in this study were high with a small number of them presenting with severe forms.

The prevalence of anxiety symptoms observed in this study amongst adolescents is higher than that seen in adolescents in the pre-COVID era. For instance, a study amongst Brazilian adolescents showed the prevalence rate of anxiety symptoms as 6.2% according to the ICD-10 classification,^22^ whilst in the United States, data from the 2016 National Survey of Children’s Health (NSCH) reported the prevalence rate of anxiety symptoms as 7.1% amongst children aged 3–17 year.^23^ Furthermore, the prevalence rate of 14% was documented in the Wuhan study^10^ and the prevalence of 18.92% was documented by Chen et al.^24^ In the same vein, the prevalence rate of depressive symptoms found in this study was much higher than 11.78% observed by Chen et al.^24^ and 14.6% obtained by Lei et al.^6^, 14.6% observed in the Wuhan study, 16.5% reported by Wang et al.^7^ and 17.3% by Rossi et al.^8^ in Italy. The studies by Lei, Wang and Rossi et al. and the Wuhan study were all carried out in the adult population. This may explain the difference in prevalence rates in their studies compared to ours. Besides, the high prevalence of anxiety in our study was mainly clustered around children with mild anxiety symptoms.

Nevertheless, the prevalence of anxiety symptoms in students of China was 43.7% and this was higher than that seen in our study. The prevalence of comorbid depressive and anxiety symptoms amongst the students noted as 31.3% in China is similar to the 33.7% of adolescents obtained in our study.^25^ Possible speculations for this similar prevalence could be because of the high population of adolescents clustering more in the middle and higher classes seen in both studies.^25^

The prevalence of severe depressive symptoms found in this study could be explained by the high burden of economic hardship caused by the lockdown during the COVID-19 pandemic in an already fragile economic situation in the country.

The emotional development of adolescents has been influenced by the COVID-19 pandemic; this is even made worse when there is an affected adult around them.^26^ Based on the World Health Organization recommendations for managing psychological and mental consequences of the COVID-19 pandemic, adolescents must be encouraged to express their fears and doubts in their ways. The high prevalence of anxiety and depressive symptoms obtained in this study is much higher than the worldwide prevalence of 6.5% documented by the Diagnostic and Statistical Manual (DSM) and International Statistical Classification of Diseases and Related Health Problems (ICD).^27^ Catastrophic and harsh economic state of the country, loss of jobs of parents of adolescents during the lockdown, increased incidence of adolescent sexual abuse during the lockdown and school closures could all account for the prevalence of both anxiety and depressive symptoms noted in this study.

In general, the prevalence of anxiety and depressive symptoms is significantly influenced by socio-cultural and socio-economic contexts.^28,29^ This could also explain the varying degrees of prevalence in each country. There is, therefore, a need to assess this in various countries and regions.

In keeping with documented reports on gender correlates of anxiety symptoms depressive symptoms spectrum, it is noted in this study that male adolescents were about twice less likely to be anxious when compared with those who were females. Anxiety symptoms and depressive symptoms affect females more than males. Metacognitive beliefs about the uncontrollability of worry are more expressed in the female gender.^30^ Bahrhami et al.^30^ documented the theory of dysphoria mood to be associated with a very high emotional state (emotional reasoning appears to characterise anxiety symptoms disorders^31^), and that rumination might mediate the associations of other risk factors for dysphoria and depressive symptoms. They opined that the higher prevalence of anxiety and depressive symptoms in female respondents may be explained by the tendency of females to ruminate and worry. There are metacognitive beliefs in rumination in people with recurrent major depressive symptoms. Women through positive metacognitive beliefs view rumination as a helpful strategy for gaining insight, identifying causes and triggers of depressive symptoms, solving problems and preventing future mistakes and failures and prioritising important tasks.^31,32^

It is shown in this study that younger adolescents are less likely to be depressed or anxious compared with older ones. A study in China also noted that older adolescents are more depressed compared with younger ones. Reasons such as the fact that older adolescents who are also in higher class face the most important challenge of their lives (the secondary school certificate examination and the university entrance examination), the COVID-19 pandemic, lockdown and school closure disrupt their normal pace of learning, with attendant increased pressure and stress.

Besides, Chinawa et al.^17^ noted that challenges of facing a new learning environment and harder and more complex curriculum in higher class could also account for this increase in prevalence of anxiety and depressive symptoms.

It is noted in this study that adolescents who had poor social support were 3.3 times more likely to be anxious when compared with those who had strong social support. Furthermore, it is seen in this study that adolescents who had moderate social support were 1.5 times more likely to be depressed when compared with those who had strong support.

Meng et al.^33^ also noted a positive effect of social support on mental health in Chinese adolescents during the outbreak of COVID-19. These findings indicate that social support is an important protective factor for the mental health of the adolescent child. The more social support adolescents receive, the better their mental status. Family members or friends reduce anxiety and depressive symptoms levels of adolescents by sharing empathy with them.^12^ Social support also improves an individual’s sense of self-esteem which culminates in mutual respect, encouragement, self-worth and fulfilment. These may help the adolescents maintain relatively stable emotions even under pressure.^34^ Poor social support could lead to depressive symptoms amongst adolescents, such as poor sleep and feeling that life is worthless, as documented in this study. Furthermore, adolescents who were more resilient were twice less likely to be depressed when compared with those who were less resilient.

The positive association of resilience in adolescents with anxiety symptoms and depressive symptoms was not in keeping with that of Sakka et al.^35^ who noted no correlation between test anxiety symptoms and resilience. The small sample size of 67 high school students and online data collection could explain this variation.

Resilience is highly related to brain maturation and biopsychosocial factors that support executive functioning in protecting against risk factors, whilst impaired resilience may exacerbate symptoms of anxiety and depressive symptoms.^36^ Chahal et al.^36^ noted that internalised symptoms in adolescence and sudden maturation of functional architecture of the brain could impair resilience and worsen anxiety and depressive symptoms in adolescents as predictors of mental health in adulthood in the Northern Swedish cohort.

Based on the result of positive associations of resilience and social support on adolescents who are depressed or anxious, cognitive behavioural therapy training and eye movement desensitisation and reprocessing (EMDR) therapies should be adopted to boost the psychological resilience of the adolescent child.^36^

It is important to note that 5.8% of college adolescents had sleep disorder during the lockdown period. The school closures and lockdown posed by the pandemic may be contributory.

Although there is a paucity of study on insomnia-related lockdown amongst adolescents, the word ‘coronasomnia’ or ‘covid-somnia’ has been used to describe insomnia-related illness amongst adults during the COVID-19 lockdown. For instance, in the UK, a study by the University of Southampton showed that the number of people experiencing coronasomnia rose from one in six to one in four, and in China, the rate rose from 14.6% to 20% during the peak lockdown. Furthermore, a high prevalence rate of clinical insomnia (40%) was observed in Italy and Greece.^37,38^

Good social support and resilience could reduce symptoms of insomnia and indirectly reduce anxiety and depressive symptoms.^39^

When we used the optimal analytic strategy of linear regression, we noted a unique association of resilience and social support. Increases in resilience correlated with increases in social support and increases in resilience also correlate with decreases in anxiety and depressive symptoms. Furthermore, increases in anxiety symptoms correlate with increases in depressive symptoms.

Some studies have shown that social support and resilience are two resources that protect individual mental health in situations of stress. Social support is key to resilience especially if it is seen to be an outcome.^40,41^

A study has also shown that social support significantly influenced the relationship between resilience and subjective well-being of some college students.^42^

## Conclusion

Resilience and good social support had a positive influence on anxiety and depressive symptoms amongst college adolescents during the lockdown in the COVID-19 pandemic despite their high prevalence rates.

### Recommendation

A good social and cultural network is very important to help adolescents cope with the huge associations created by the COVID-19 pandemic during the lockdown. Alternative measures other than lockdown and school closures may also be necessary to curb the high prevalence rates of anxiety and depressive symptoms amongst adolescents in the COVID-19 lockdown period.

### Limitations

This study was executed in the south-eastern part of the country, and as such, the finding may not apply to the greater population of the country. A nationwide study may help to make this study more elaborate.
